# Antibacterial Effect of Oregano Essential Oil against *Vibrio vulnificus* and Its Mechanism

**DOI:** 10.3390/foods11030403

**Published:** 2022-01-30

**Authors:** Kunyao Luo, Pengyu Zhao, Yifei He, Shengnan Kang, Chenyu Shen, Shuo Wang, Meixian Guo, Lehui Wang, Chao Shi

**Affiliations:** College of Food Science and Engineering, Northwest A&F University, 20 Xinong Road, Yangling, Xianyang 712100, China; luokunyao725@163.com (K.L.); zhaopengyu1999@163.com (P.Z.); 6210112025@stu.jiangnan.edu.cn (Y.H.); kangshengnan@nwafu.edu.cn (S.K.); shenchenyu@nwafu.edu.cn (C.S.); wangshuoddu@126.com (S.W.); guomeixian@nwafu.edu.cn (M.G.); wanglehui@nwafu.edu.cn (L.W.)

**Keywords:** antimicrobial activity, oregano essential oil, *Vibrio vulnificus*, membrane damage, reactive oxygen species, comet assay

## Abstract

Oregano essential oil (OEO) is an effective natural antibacterial agent, but its antibacterial activity against *Vibrio vulnificus* has not been widely studied. The aim of this study was to investigate the inhibitory effect and germicidal activity of OEO on *V. vulnificus* and its possible inhibition mechanism. The minimum inhibitory concentrations (MICs) and minimum bactericidal concentrations (MBCs) of OEO against four *V. vulnificus* strains (ATCC 27562 and three isolates from seafoods) were from 0.06 to 0.15 μL/mL. Compared with untreated bacteria, OEO reduced the concentration of intracellular adenosine triphosphate (ATP), hyperpolarized the cell membrane, increased the level of reactive oxygen species (ROS), and increased the concentration of intracellular malondialdehyde (MDA), but there was no obvious DNA damage at the OEO test concentration. It was indicated that OEO inactivated *V. vulnificus* by generating ROS which caused lipid peroxidation of cell membranes, thereby reducing the permeability and integrity of cell membranes and causing morphological changes to cells, but there was no obvious damage to DNA. In addition, OEO could effectively kill *V. vulnificus* in oysters at 25 °C, and the number of bacteria decreased by 48.2% after 0.09% OEO treatment for 10 h. The good inhibitory effect and bactericidal activity of OEO showed in this study, and the economy and security of OEO make it possible to apply OEO to control *V. vulnificus* contamination in oysters and other seafoods.

## 1. Introduction

*Vibrio vulnificus* is a Gram-negative food-borne opportunistic pathogen with slightly curved shape, extreme single flagella, low salinity and [1]. *V. vulnificus* widely exists in estuaries and coastal areas all over the world, and is often isolated from oysters, fish, shrimp and other aquatic products [2]. *V. vulnificus* is the leading cause of seafood related disease and is increasing in prevalence globally [3]. Humans can be infected with *V. vulnificus* by ingesting raw or undercooked seafood or by contacting contaminated seawater in wounds. Food intake can lead to gastroenteritis and sepsis, and wound contact can lead to tissue necrosis near the wound [4]. The mortality rates are 50% and 17%, respectively [5]. At present, *V. vulnificus* infection cases often have acute onset, high mortality, high disability rate and great harm, which has important public health significance [6] that has attracted great attention of Australia, Japan, and other coastal countries and regions [7].

It is very important to employ a safe and effective alternative method to control *V. vulnificus* in oyster, shrimp, crab and other seafood, so as to reduce the risk of foodborne diseases of *V. vulnificus* in these foods. Several prevention and control methods have been proposed, including gamma irradiation [8], modified atmosphere packaging [9], and chemical preservatives [10]. These methods have their own advantages [9], but they also have limitations. For example, the requirements of the gamma irradiation working conditions are strict, the equipment is complex, and the cost is high [11]. Moreover, the effectiveness of fresh-keeping packaging technology is closely related to processing procedures and the storage conditions of in-process products [12]. Chemical preservatives are potentially toxic and may induce adverse health effects [13]. Therefore, it is extremely important to find a safe and effective alternative method to control *V. vulnificus*. In recent years, a variety of natural, environmental friendly and safe plant essential oils have attracted extensive attention because of their good anti-bacterial effects [14].

Oregano (*Origanum vulgare* L.) is an aromatic plant with a wide distribution throughout the Mediterranean area and Asia [15]. Oregano essential oil (OEO) is a complex mixture of compounds. The composition of OEO commonly includes terpenes, generally mono- and sesquiterpenes. The principal terpenes identified in the different species of oregano are carvacrol, thymol, γ-terpinene and p-cymene. Terpinen-4-ol, linalool, β-myrcene, trans-sabinene hydrate, and β-caryophyllene are also present [16]. OEO is classified as “generally recognized as safe” (GRAS) by the Food and Drug Administration [17], and classified as a food additive by the European Union [18]. In view of the characteristics of OEO, such as rapid action, no residue, difficulty producing drug resistance, low phytotoxicity [19] and harmlessness to mammals [19,20], it has potential applications to be used as an antibacterial agent in marine products. However, the antibacterial activities of OEO against *V. vulnificus* have rarely been reported.

Based on the above factors, the purpose of this study was to study the antibacterial activity and mode of action of OEO against *V. vulnificus*. In order to achieve this goal, this experiment first evaluated the inhibitory effect of OEO against *V. vulnificus*, and then explored its antibacterial mechanism on *V. vulnificus* by detecting its effects on the growth curve, intracellular ATP, membrane potential change, reactive oxygen species (ROS), malondialdehyde (MDA), DNA damage, cell membrane integrity and cell morphology. In addition, the antibacterial activity of OEO against *V. vulnificus* in contaminated oyster samples was evaluated.

## 2. Materials and Methods

### 2.1. Reagents

OEO (density 0.939 g/mL at 25 °C, CAS 8007-11-2) and dimethyl sulfoxide (DMSO; analytical grade) were purchased from Sigma-Aldrich (Shanghai, China). Alkaline peptone water (APW) and 3% NaCl tryptone soya agar (TSA) were purchased from Land Bridge Technology Co. (Beijing, China). All other chemicals and reagents were of analytical grade. Before each assay, OEO was dissolved in DMSO and vortexed for 30 s at room temperature. The final concentration of DMSO in all of the sample solutions (treatment and control samples) was 0.1% (*v*/*v*), which has no apparent effect on the growth of *V. vulnificus*.

### 2.2. Bacterial Strains and Culture Conditions

*V. vulnificus* strain ATCC 27562 was purchased from the American Type Culture Collection (ATCC, Manassas, VA, USA). The other three *V. vulnificus* strains (VV 2, VV 15, VV 20) were obtained from the Chinese University of Hong Kong laboratory and were originally isolated from seafood collected from supermarkets. Only *V. vulnificus* ATCC 27562 was used for antibacterial mechanism analysis. ATCC 27562 and three isolates (VV 2, VV 15 and VV 20) were used for the experiment in fresh oysters. All strains were stored in 3% (*w*/*v*) NaCl APW with 20% (*v*/*v*) glycerol at −80 °C. Stock cultures were streaked in 3% NaCl TSA and grown at 37 °C for 14 h prior to each experiment. A loop of the strain was then inoculated into 30 mL APW and cultured in a shaking table (130 rpm) at 37 °C for 16 h to the late logarithmic growth stage. The bacterial culture was washed twice with phosphate-buffered saline (PBS, pH 7.2), then subsequently adjusted to OD_600 nm_ = 0.5 (~4 × 10^8^ CFU/mL) with APW or PBS and standby.

### 2.3. Determination of MICs and MBCs

The MICs of OEO against *V*. *vulnificus* strains was measured using the broth microdilution method as described by Clinical and Laboratory Standards Institute guidelines [21], with minor modifications. Each well of a 96-well plate was inoculated with 200 μL of diluted bacterial strain culture, with a final bacterial concentration of approximately 5 × 10^5^ CFU/mL. OEO was added to each well to achieve final concentrations of 0.48, 0.24, 0.12, 0.06, 0.03, 0.015 or 0 (control) μL/mL. APW broth containing 0.1% DMSO was used as the blank background sample. APW containing 0.1% DMSO dissolved with 0.1 mg/mL chloramphenicol was used as the positive control. The MICs were defined as the lowest concentration of OEO that resulted in no visible *V. vulnificus* growth within 24 h. In order to determine MBCs, 100 μL of bacterial solution was taken from each well showing inhibition, plated on 3% NaCl TSA agar and cultured for 48 h, and the lowest antimicrobial concentration that did not allow any bacterial growth was recorded as the MBC.

### 2.4. Growth Curves

The method was followed to determine growth curve as described by Shi et al. [22], with slight modification. The bacterial suspension was prepared according to the method described in Section 2.2. OEO was added to a bacterial suspension which was approximately diluted to 4 × 10^6^ CFU/mL to obtain the final concentrations of 1/16MIC, 1/8MIC, 1/4MIC, 1/2MIC and MIC. *V. vulnificus* ATCC 27562 with or without OEO was incubated at 37 °C and cell growth was determined by measuring OD_600 nm_ every 1 h using a multimode plate reader (Model 680; Bio-Rad Laboratories, Hercules, CA, USA) for 24 h. Three independent replicates of each experiment were performed.

### 2.5. Antibacterial Effect of OEO on V. vulnificus in APW

The bactericidal activity of OEO against *V. vulnificus* ATCC 27562 was tested using the plate count method described by Ashrafudoulla et al. [23], with some modification. The bacterial suspension was prepared according to the method described in Section 2.2 approximately diluted to 10^6^ CFU/mL, and then mixed with OEO to achieve the final concentrations of 0 (control), 1/4MIC, 1/2MIC, and MIC. The prepared samples were incubated at 37 °C for 0, 20, 40, 60, 90 and 120 min. Subsequently, 100 μL of each sample was spread onto 3% NaCl TSA plate and incubated at 37 °C for 14 h. This experiment was repeated three times independently.

### 2.6. Intracellular ATP Concentrations

Intracellular ATP concentration determination was carried out as described by Sanchez et al. [24], with slight modification. The bacterial suspension (~4 × 10^8^ CFU/mL) was prepared according to the method described in Section 2.2, and OEO was added to bacterial suspension to achieve final concentrations of 0 (control), MIC, and 2MIC, respectively. The samples were cultured at 37 °C for 30 min, and then the cells were lysed by sonication on ice. After centrifugation at 5000× *g* for 5 min, the supernatant was gathered and stored on ice to prevent ATP loss. Intracellular ATP was measured with an ATP assay kit (Beyotime Bioengineering Institute, Shanghai, China). Briefly, the mixtures (125 μL ATP assay working solution and 125 μL supernatant/125 μL ATP gradient standard solution) were added to white, opaque, 96-well microtiter plates, and the supernatant fluorescence values were measured with a microplate reader (SpectraMax M2, Molecular Devices, San Jose, CA, USA). A standard curve was constructed based on ATP standard solutions (0.01, 0.10, 1.00 and 10.00 μM) and their corresponding luminescence values to calculate the concentration of ATP in the sample cells. Three independent replicates were performed for each experiment.

### 2.7. Membrane Potentials

The measurement method of membrane potential refers to that described by Yang et al. [25]. Briefly, the bacterial suspension (~4 × 10^8^ CFU/mL) was prepared according to the method described in Section 2.2. The bacterial suspension prepared in Section 2.2 was inoculated into a black 96-well plate, and then a fluorescent dye (DiBAC_4_(3)) was added to each well to a final concentration of 1 μmol/L. After sample incubation at 37 °C for 30 min, OEO was added to final concentrations of 0 (control), MIC and 2MIC. Fluorescence measured by a fluorescence microplate reader (Spectra Max M2) at excitation and emission wavelengths of 492 and 515 nm were measured at 1, 4, 8 and 12 min, respectively. Membrane potential was illustrated in terms of relative fluorescence units (RFUs; RFU = (FU_treatment_ − FU_background_) − (FU_control_ − FU_background_)), with three independent replicates included in each experiment.

### 2.8. Detection of Reactive Oxygen Species (ROS)

According to Shivaprasad et al. [26], the fluorescent molecule dichlorodihydrofluorescein diacetate (DCFH-DA; Beyotime Institute of Bio-technology, Shanghai, China) was used to determine intracellular ROS levels in bacteria. The bacterial suspensions (~4 × 10^8^ CFU/mL) prepared according to Section 2.2 were added OEO (at final concentrations of 0 (control), 1/4MIC, 1/2MIC and MIC) and then incubated at 37 °C for 15 min. Next, the mixture was incubated with DCFH-DA (final concentration 5 μM) at 37 °C for 15 min. After incubation, cells were washed twice with PBS. The relative fluorescence intensity was measured (excitation 488 nm, emission 525 nm) by a multimode microplate reader (Spectra Max M2), and the data were normalized by the number of survival bacteria in the cell suspensions.

### 2.9. Malondialdehyde (MDA) Content Assay

The determination of MDA content was carried out as described by Cao et al. [27], with slight modification. The bacterial suspension was prepared according to the method described in Section 2.2 and approximately diluted to 1 × 10^6^ CFU/mL. Cultures of *V*. *vulnificus* ATCC 27562 with and without OEO treatment (0 (control), 1/4MIC, 1/2MIC and MIC) were incubated at 37 °C for 30 min. After centrifugation to collect bacteria, the extract was added according to the proportion of 5 million bacteria to 1 mL. Subsequently, the samples were centrifuged for 10 min at 8000× *g,* and the supernatants were collected. The contents of MDA in the supernatants were determined according to the instructions in the lipid peroxidation MDA assay kit (Solarbio Life Sciences, Beijing, China) by using a multimode microplate reader (Spectra Max M2) at 450 nm, 532 nm and 600 nm. MDA content (nmol/mL) = 5 × (12.9 × (ΔA_532 nm_ − ΔA_600 nm_) − 2.58 × ΔA_450 nm_).

### 2.10. DNA Comet Assay

The comet assay for detecting DNA damage of *V. vulnificus* caused by OEO as described by Kim et al. [28] and slightly modified. Briefly, *V. vulnificus* (~4 × 10^8^ CFU/mL) was treated with OEO (0 (control), MIC, 2MIC and 4MIC) at 37 °C for 2 h. The treated samples (75 μL) were mixed with 90 mL Comet Agarose, 5 mg/mL RNase A solution (SigmaeAldrich), 0.25% N-Lauroylsarcosine sodium salt solution (SigmaeAldrich) and 0.5 mg/mL lysozyme (Sigmae Aldrich). Then, 75 μL of the mixture was placed on a Comet slide and refrigerated at 4 °C in the dark for 15 min to solidify on the slide. Slides were immersed in 1× lysis buffer (pH 10) for 45 min and then in alkaline solution for 30 min in the dark at 4 °C. Subsequently, the slides were electrophoresed in alkaline running buffer at 12 V and 100 mA for 20 min. After electrophoresis, the slides were soaked and rinsed three times in distilled water, and then placed in pre-cooled 70% (*v*/*v*) ethanol. After 5 min, the ethanol was blotted dry and the slides were allowed to air dry naturally. Finally, 100 μL of Vista Green DNA dye was added to each well of the slides, and the samples were visualized by fluorescence microscopy (LECIA DM6 B; Lecia, Wetzlar, Germany) under a 100× oil microscope.

### 2.11. Confocal Laser Scanning Microscopy (CLSM) Observations

The cell membrane integrity of *V. vulnificus* ATCC 27562 was determined using two nucleic acid dyes, SYTO 9 (green) and propidium iodide (PI, red), as described by Qi et al. [29], with minor modifications. Briefly, *V. vulnificus* (~2 × 10^8^ CFU/mL) was treated with OEO (0 (control), MIC, 2MIC and 4MIC) and incubated at 37 °C for 30 min. The cell suspension was then centrifuged at 10,000× *g* for 5 min and resuspended in 200 μL of 0.85% (*m*/*v*) NaCl. Then, 0.5 μL of a mixed dye of PI and SYTO 9 was added to the bacterial resuspension solution and incubated for 5 min at room temperature. Untreated *V. vulnificus* served as a control sample. Finally, 5 μL of the sample was placed on a glass slide and observed at 400× magnification using a confocal laser scanning microscope (A1; Nikon, Tokyo, Japan).

### 2.12. Field-Emission Scanning Electron Microscopy (FESEM) Observations

The FESEM assay was performed according to the method previously published by Tian et al. with slight modifications [30]. Bacterial suspensions (~4 × 10^8^ CFU/mL) obtained according to the method described in Section 2.2 were treated with OEO (0, MIC, 2MIC and 4MIC). 

Then after 2 h incubation at 37 °C, the cells were centrifuged (5000× *g*, 10 min, 4 °C) and washed twice with PBS. Afterwards, the cells were maintained in 2.5% (*v*/*v*) glutaraldehyde at 4 °C to fix the cells. The cells were harvested by centrifugation after 12 h and dehydrated with 30%, 50%, 70%, 80%, 90% and 100% ethanol for 10 min, shaken at each concentration. Finally, the samples were mounted on a FESEM stand for sputter-coated gold under vacuum and examined under a scanning electron microscope (S-4800; Hitachi, Tokyo, Japan; 20,000× magnification).

### 2.13. Antibacterial Effect of OEO against V. vulnificus in Fresh Artificially Contaminated Oysters Model

Fresh oysters were purchased from the local supermarket (Haoyouduo, Xianyang, China). Suspensions of *V. vulnificus* strains ATCC 27562, VV2, VV15 and VV20 were prepared as described in Section 2.2 (~4 × 10^8^ CFU/mL). Later, equal volumes of the four bacterial suspensions were then mixed for use in the following assays. Fresh oysters cleaned under sterile conditions were immersed in 3% (*v*/*v*) NaClO for 20 min to remove the pre-existing bacteria. The oysters were then washed twice with sterile water to remove residual NaClO. The oyster meat was mixed with an equal amount of PBS and stirred to obtain an oyster homogenate (dilution 1:2). The mixed bacteria suspension was added into the oyster homogenate so that the concentration of *V. vulnificus* in the oysters was 10^6^ CFU/g. Then, OEO was added to aliquots of a mixed solution of bacterial suspension and oyster homogenate to make final concentrations of OEO of 0 (control), 0.03%, 0.06% and 0.09% (*v*/*v*). The samples were incubated at 25 °C and collected at 0, 2, 4, 6, 8 and 10 h, then diluted with PBS and spread on 3% NaCl TSA plates and counted. (The results were expressed as log CFU/g.)

### 2.14. Statistical Analysis

All experiments were performed in triplicate independently. All data are presented as the mean ± standard deviation (SD); differences between means were tested using a Student’s *t*-test and analyzed using IBM SPSS software (version 19.0; SPSS Inc., Chicago, IL, USA). *p* < 0.05 was considered significant, *p* < 0.01 was considered extremely significant.

## 3. Results

### 3.1. MIC and MBC of OEO against V. vulnificus

OEO showed inhibitory effects against all four *V. vulnificus* strains tested (Table 1). Overall, MICs ranged from 0.06 to 0.15 μL/mL, with OEO showing the greatest sensitivity to strain ATCC 27562 (MIC = 0.06 μL/mL). The MBC values of all tested strains in this study were identical to the MIC values. Only *V. vulnificus* ATCC 27562 was selected for further study.

### 3.2. Effect of OEO on V. vulnificus Growth

**Figure 1:**
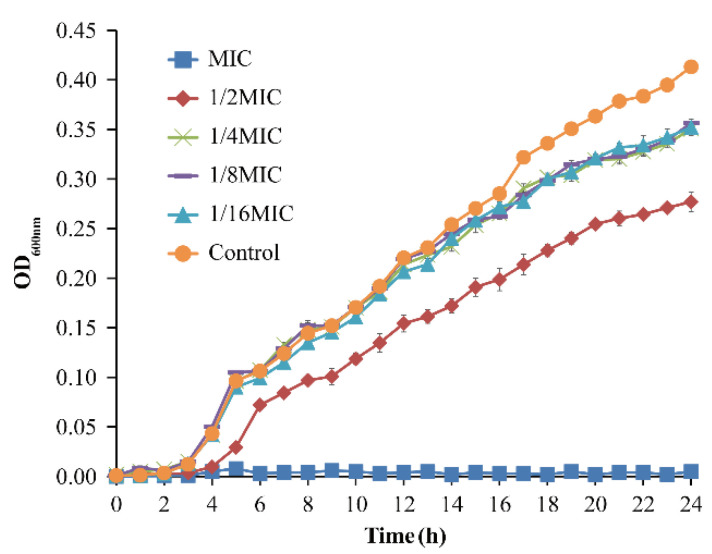
Growth curves for *V. vulnificus* ATCC 27562 in alkaline peptone water (APW) with various concentrations of OEO. (OD is the abbreviation of optical density).

Figure 1 shows the effect of OEO from 1/16MIC to MIC concentration on the growth of *V. vulnificus* ATCC 27562. OEO at the MIC completely inhibited the growth of *V. vulnificus* in APW. The lag phase of *V. vulnificus* was prolonged when treated with at 1/2MIC OEO. OEO at levels below 1/4MIC had no significant effects on the growth of *V. vulnificus*.

### 3.3. Antibacterial Activity of OEO toward V. vulnificus in APW

**Figure 2:**
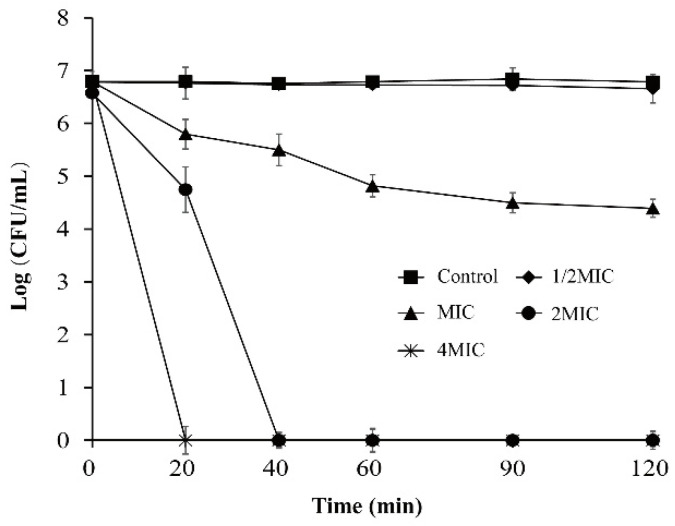
Effect of OEO on *V. vulnificus* ATCC 27562 populations in APW.

The antibacterial effect of OEO on *V. vulnificus* in APW is shown in Figure 2. The initial viable count was about 6.8 log CFU/mL. Bacteria treated without OEO did not change significantly within 120 min. The 1/2MIC treatment group had no obvious inhibitory effect on *V. vulnificus*. When *V. vulnificus* was exposed to OEO at MIC, the bacterial count continuously decreased to 4.39 ± 0.17 log CFU/mL within 120 min. Under the OEO treatments of 2MIC and 4MIC, the number of bacteria decreased to below the detection limit within 40 min and 20 min, respectively (the detection limit was 1 CFU/mL).

### 3.4. Effect of OEO on Intracellular ATP Concentration

**Figure 3:**
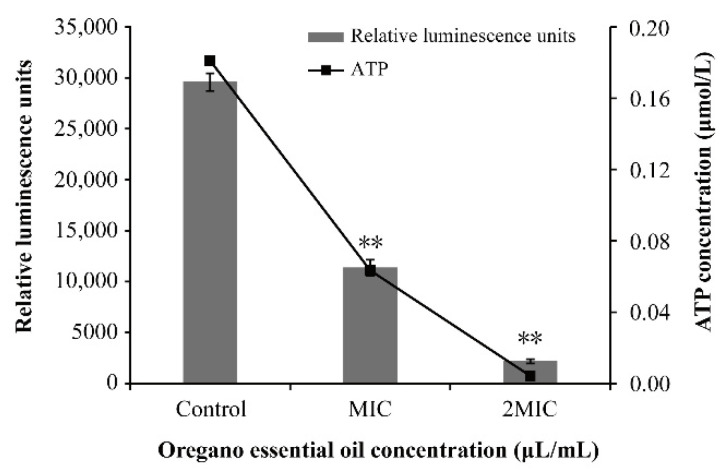
Effects of OEO treatment on intracellular ATP content of *V. vulnificus* ATCC 27562 (** *p* < 0.01).

There was a good linearity between relative luminescence units and ATP concentration (y = 154,889x + 1537; R^2^ = 0.999, the standard curve was shown in the Figure S1). The level of intracellular ATP of *V. vulnificus* decreased extremely significantly (*p* < 0.01) as the OEO concentration increased (Figure 3). The original ATP concentration of *V. vulnificus* ATCC 27562 was 0.181 ± 0.002 μmol/L. The addition of OEO at MIC and 2MIC caused ATP concentrations to be reduced to 0.063 ± 0.004 and 0.004 ± 0.001 μmol/L respectively.

### 3.5. Effect of OEO on Membrane Potential

**Figure 4:**
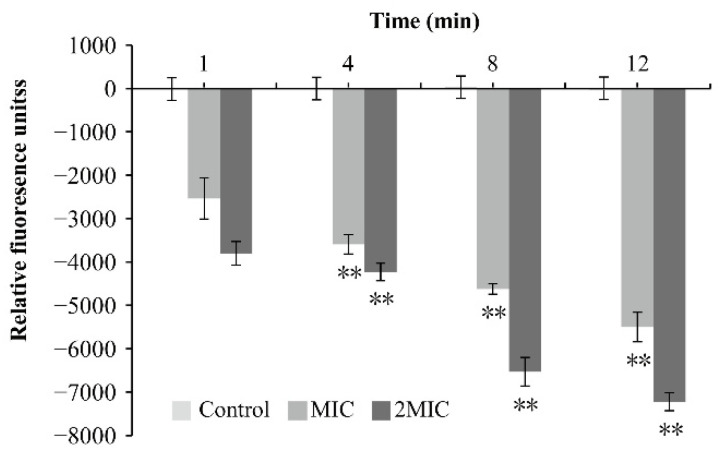
Effect of OEO on the cell membrane potential of *V. vulnificus* ATCC 27562 (** *p* < 0.01).

The effects of OEO on the membrane potential of *V. vulnificus* ATCC 27562 are shown in Figure 4. Hyperpolarization (indicated by negative relative fluorescence values) was observed in *V. vulnificus* ATCC 27562 samples treated with OEO at the concentrations of 0 (control), MIC, and 2MIC, and the relative fluorescence intensity increased negatively, which was extremely significant when compared with the control sample (*p* < 0.01). Moreover, as the concentration of OEO increased, the hyperpolarization of the cell membrane of *V. vulnificus* increased, and with the increase of time, the cell membrane hyperpolarization also showed an increasing trend.

### 3.6. Effect of OEO on Intracellular ROS Generation

**Figure 5:**
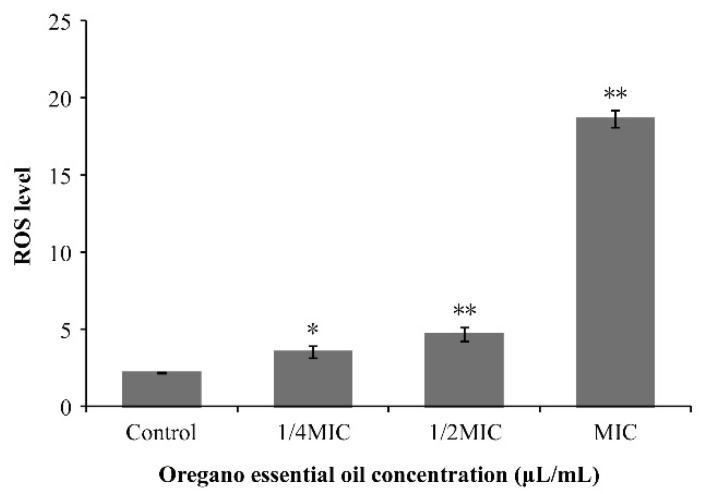
Intracellular reactive oxygen species (ROS) level in *V. vulnificus* ATCC 27562 treated with OEO (* *p* < 0.05, ** *p* < 0.01).

Figure 5 shows ROS level in *V. vulnificus* ATCC 27562 treated with OEO. As shown in the figure, the ROS level in the *V. vulnificus* cells increase with the enhanced concentrations of OEO. The ROS level of *V. vulnificus* cells was 2.15 ± 0.04 in the control sample, while the levels cells treated with OEO at 1/4MIC, 1/2MIC and MIC were 3.51 ± 0.39, 4.64 ± 0.45, 18.62 ± 0.55 after 30 min, respectively.

### 3.7. Effect of OEO on Intracellular MDA Content of V. vulnificus

**Figure 6:**
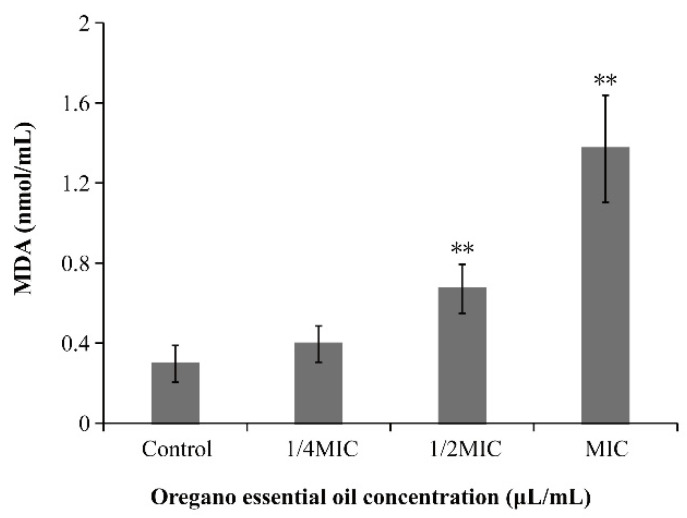
Intracellular malondialdehyde (MDA) in *V. vulnificus* ATCC 27562 treated with OEO (** *p* < 0.01).

The production of MDA was analyzed by the thiobarbituric acid method. As shown in Figure 6, compared with the control sample, the intracellular malondialdehyde level of *V. vulnificus* treated with OEO increased in a concentration-dependent manner. When *V. vulnificus* was treated with OEO at the concentration of 1/2MIC and MIC, the content of intracellular MDA increased significantly from 0.30 ± 0.09 nmol/mL to 0.67 ± 0.12 nmol/mL and 1.37 ± 0.27 nmol/mL, respectively (*p* < 0.01).

### 3.8. Effect of OEO on DNA Damage

**Figure 7:**
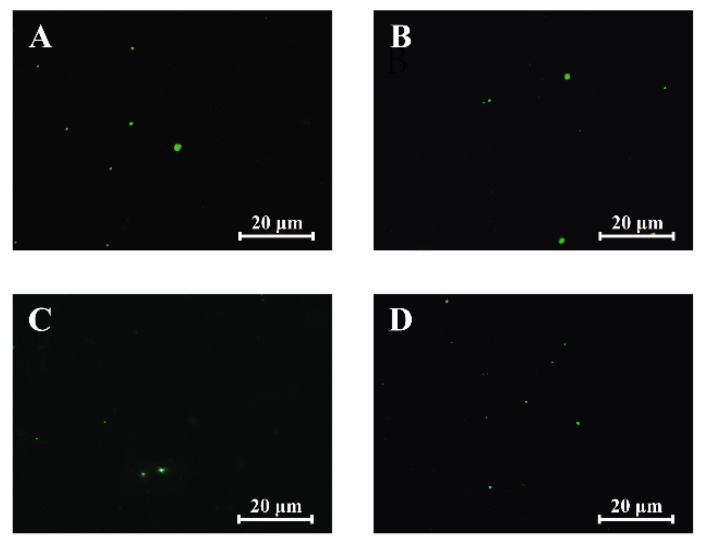
Representative figures of the comet assay of *V. vulnificus* ATCC 27562 without (**A**) and with treatment with OEO with MIC (**B**), 2MIC (**C**), and 4MIC (**D**).

The comet assay was used to study the effect of different concentrations of OEO on DNA damage. As shown in Figure 7, no tail (comet) was observed in both OEO treated and non OEO treated cells regardless of OEO concentration, indicating that OEO treatment did not cause DNA fragmentation of *V. vulnificus*.

### 3.9. CLSM-Based Observations of Cell Membrane Injury

**Figure 8:**
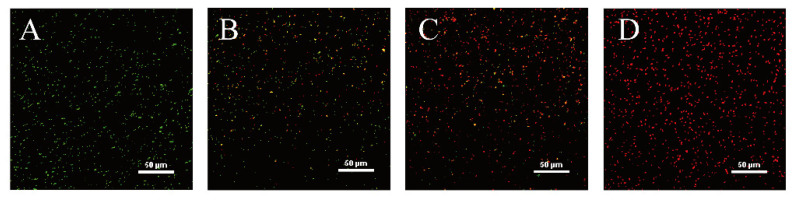
CLSM of *V. vulnificus* ATCC 27562 without (**A**) and with treatment with OEO with MIC (**B**), 2MIC (**C**), and 4MIC (**D**).

As shown in Figure 8, the control sample appeared almost exclusively contained green, indicating that the cell membranes of the untreated cells were intact (Figure 8A). In comparison, in the cells treated with OEO at MIC a small amount of red fluorescence appeared (Figure 8B), and the red fluorescence was more obviously observed in the 2MIC sample (Figure 8C). In the 4MIC sample, an overwhelming fraction of the treated *V. vulnificus* cells emitted yellow or red fluorescence with little green fluorescence (Figure 8D). These results suggested that the integrity of *V. vulnificus* cell membranes were decreased by OEO in a concentration-dependent manner. 

### 3.10. FESEM-Based Observations of Cell Morphology

**Figure 9:**
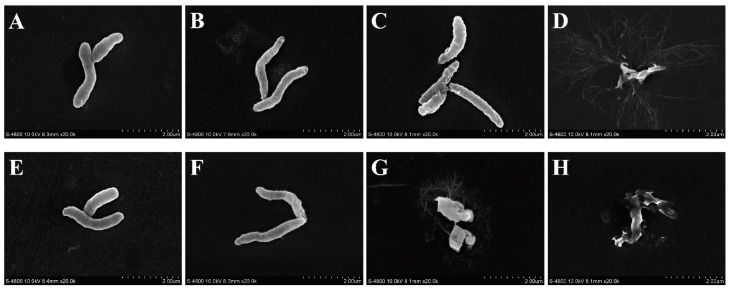
Field-emission scanning electron micrographs of *V. vulnificus* ATCC 27562. Untreated bacterial cells at 2 h (**A**) and 4 h (**E**) post-inoculation. Bacterial cells treated with OEO at MIC for 2 h (**B**) and 4 h (**F**). Bacterial cells treated with OEO at 2MIC for 2 h (**C**) and 4 h (**G**). Bacterial cells treated with OEO at 4MIC for 2 h (**D**) and 4 h (**H**).

FESEM images of *V. vulnificus* ATCC 27562 cells treated with OEO are shown in Figure 9. The *V. vulnificus* without OEO treatment was arc-shaped, long, round and full, with a smooth surface (Figure 9A,E). After treatment with MIC OEO for 2 h and 4 h, the surface of *V. vulnificus* was wrinkled (Figure 9B,F). After treatment with 2MIC OEO for 2 h, the cell appeared to undergo more serious shrinkage and collapse (Figure 9C). After treatment with 2MIC OEO for 4 h (Figure 9G), the cell broke up and lost its original form. After treatment with 4MIC OEO for 2 h and 4 h, the bacteria completely broke down and became fragments (Figure 9D,H). It can be seen that OEO can cause serious damage to the morphology of *V. vulnificus*, and with the increase of OEO concentration and treatment time, the damage degree to cell morphology is greater.

### 3.11. Antibacterial Effect of OEO on V. vulnificus in Fresh Oysters

**Figure 10:**
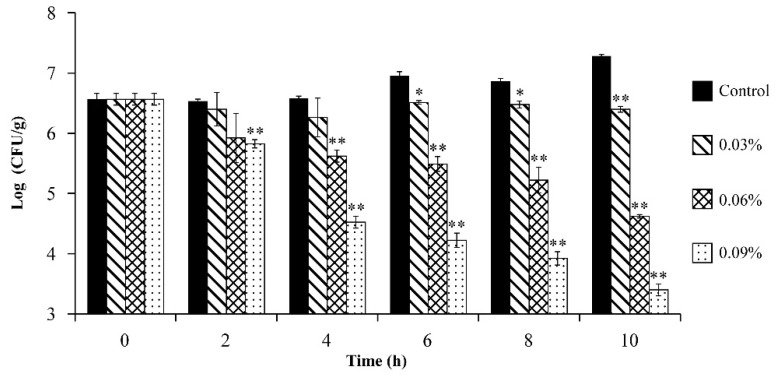
Inhibitory effects of OEO against *V. vulnificus* in oysters at 25 °C. Bars represent the standard deviation (*n* = 3). * *p* < 0.05, ** *p* < 0.01.

An artificially contaminated oyster model was constructed to evaluate the bacteriostatic effect of OEO against *V. vulnificus* in food medium (Figure 10). At 0 h, *V. vulnificus* was 6.56 ± 0.09 log CFU/g in all samples. After 10 h, the number of *V. vulnificus* in oysters without OEO increased by 10.9%. Meanwhile, treatment with OEO at concentrations of 0.03%, 0.06%, and 0.09% (*v*/*v*) reduced the total *V. vulnificus* counts in fresh oysters to 6.39 ± 0.05, 4.16 ± 0.03, and 3.39 ± 0.10 log CFU/g, respectively. Among them, 0.06% and 0.09% (*v*/*v*) treatments reduced the number of bacteria by 29.6% and 48.2% after 10 h.

## 4. Discussion

In this study, OEO exhibited inhibitory activity against four strains of *V. vulnificus* with MIC values ranging from 0.06 to 0.15 μL/mL (50 to 140 μg/mL) (Table 1). The inhibitory effect of some natural compounds against *V. vulnificus* has also been reported in previous studies. Navarro-Navarro et al. [31] determined that the MIC of Sonoran propolis against *V. vulnificus* was 50 μg/mL, while the MIC of thyme essential oil was 190 μg/mL [32]. In addition, Ozogul et al. [33] determined that the MIC of grapefruit peel essential oil against *V. vulnificus* was 25,000 μg/mL. It is worth mentioning that, in the study of Dominguez-Borbor et al. [34], OEO was effective at inhibiting quorum sensing mediated processes in *V. vulnificus* at sublethal concentrations (1.0 μg/L). Based on the above information, in this study, the antibacterial activity of OEO against *V. vulnificus* was superior to most of these natural compounds in the literature. Early in our study, the MIC of carvacrol, the main component of OEO, against *V. vulnificus* ATCC 27562 was determined to be 50 μg/mL. Except carvacrol, OEO contains a variety of antibacterial active components, and the combination of two or more components may produce synergistic effects and improve the antibacterial activity [35]. Compared with carvacrol, OEO can show good antibacterial ability without extraction and purification. This relatively low cost and good antibacterial ability make the application of OEO more competitive in the market.

ATP is one of the most important energy-related molecules in bacteria and is closely related to the cellular function, growth and survival of microorganisms [36]. The results showed that OEO extremely significantly reduced the intracellular ATP concentration of *V. vulnificus* compared with the control sample (*p* < 0.01) (Figure 3). Similarly, Guo et al. [37] found that the intracellular ATP concentration of *Cronobacter sakazakii* treated with 0.4 mg/mL coenzyme Q0 decreased extremely significantly after 20 min (*p* < 0.01). The decrease of intracellular ATP concentration may be caused by the increase of membrane permeability after treatment with natural products, which leads to the release of ATP [38]. Moreover, *V. vulnificus* may change the existing active system to cope with environmental pressure when treated with OEO, which have been shown to affect the steady-state changes of the ATP secretion efflux pump [39] or the ATP secretion system [40].

Membrane potential is closely related to the function of bacteria, and is one of the important targets of antibacterial effect of natural products [41]. In this study, OEO induced membrane hyperpolarization of *V. vulnificus* cells in a concentration- and time-dependent manner (Figure 4). In previous studies, Bot and Prodan [42] discovered that carvacrol caused hyperpolarization of *Escherichia coli* cell membranes, and a similar phenomenon was found of citral against *C. sakazakii* [43]. Previous studies have shown that cell membrane hyperpolarization may be related to the change of pH value and the movement of sodium and potassium ions [37]. The change of membrane potential and the decrease of ATP concentration reflected that the membrane dysfunction and membrane permeability of *V. vulnificus* were changed by OEO treatment.

ROS is a normal product of cell metabolism and plays an important role in cell signal transduction and tissue homeostasis [44]. However, a high level of ROS may cause DNA damage and intracellular peroxidation of lipids, eventually leading to cell damage [45]. In this study, OEO significantly increased the ROS level of *V. vulnificus* in a concentration-dependent manner (Figure 5). Similarly, Li et al. [46] found that epigallocatechin gallate, the main polyphenol component in tea, killed *V. mimicus* by increasing the production of intracellular ROS. Likewise, limonene can induce a large number of hydroxyl radicals, thereby improving the level of ROS in *Penicillium digitatum* spores [47]. Vatansever et al. [44] believe that excessive ROS will destroy the defense systems of superoxide dismutase (SOD) and catalase (CAT) in bacteria. Therefore, the increase of ROS levels in *V. vulnificus* treated with OEO may be caused by the large production of intracellular radicals and the activity of radical scavenging system is thus decreased.

The free radical chain reaction of fat peroxidation is easily triggered because of the large number of polyunsaturated fatty acids in cell membranes [48]. MDA is one of the end products of lipid peroxidation, and can reflect the degree of lipid peroxidation of bacterial cell membrane. In this study, the increase to the intracellular MDA content of *V. vulnificus* treated with OEO was consistent with the increased trend of ROS (Figure 6). Similarly, Yang et al. [47] found that the quantitative MDA level of Carbapenemase-producing *Klebsiella pneumoniae* treated by linalyl anthranilate was significantly higher than those of untreated cells, and this trend was also found in the change of the ROS level. Moreover, Ajiboye et al. [49] considered that the increase of MDA in catechin treated *E. coli*, *Pseudomonas aeruginosa* and *Staphylococcus aureus* indicates that the cells are attacked by macromolecules, which may have been due to lipid peroxidation caused by enhanced production of reactive oxygen species. According to the analysis of ROS in this study, the increase to the intracellular MDA content of *V. vulnificus* may have been due to lipid peroxidation caused by excessive ROS. In addition, because lipids are the main component of cell membranes, the treatment of OEO may change the physical properties of bacterial cell membranes, destroy their integrity, and may lead to the final death of bacteria.

Excessive ROS production above basal levels can impair and oxidatively damage DNA, thus affecting biological development and life function operation, eventually leading to cell death [44]. In this study, no DNA tailing was observed in *V. vulnificus* treated with OEO at the concentrations of MIC, 2MIC and 4MIC, indicating that the bacterial DNA was not damaged (Figure 7). Similarly, from the treatment of *E. coli* O157:H7, *Salmonella* Typhimurium, and *Shigella sonnei* with a 405 ± 5 nm light emitting diode at a dose of 486 J/cm^2^, we did not observe a difference of DNA tailing and total genomic DNA, indicating that bacterial DNA was not damaged [28]. The ROS can first attack both the sugar moieties and the base, and then cause DNA breakage [50]. Further, the damaged DNA may also result in DNA breakage caused by the oxidative destruction of deoxyribose residues or replication forks [28]. In this study, the reason why *V. vulnificus* treated with OEO did not show tailing may have been that the low concentration of OEO cannot produce enough ROS to affect the bacterial DNA.

With an appropriate mixture of the SYTO 9 and propidium iodide stains, bacteria with intact cell membranes stained fluorescent green, whereas bacteria with damaged membranes stained fluorescent red, as observed by CLSM. This study confirmed that the red fluorescence increased significantly after OEO treatment, indicating that the membrane integrity of *V. vulnificus* was decreased (Figure 8). Similarly, Liu et al. [51] and Tian et al. [30] used the same method to prove that the cell membranes of *Listeria monocytogenes* treated with bifidocin A and *Enterobacter sakazakii* treated with thymol were both destroyed. In this study, when *V. vulnificus* was treated with OEO, the change of ATP and membrane potential hyperpolarization showed the turbulence of cell membrane permeability, while the result of MDA showed that ROS induced lipid peroxidation destroyed the integrity of cell membrane. Therefore, CLSM further verified the destruction of *V. vulnificus* cell membrane under the treatment of OEO.

The FESEM analysis of this study showed that *V. vulnificus* cells treated with OEO became shriveled, and the cells in high concentration OEO group even broke up (Figure 9). Similar to the results of this study, Cui et al. [52] treated Meticillin-resistant *Staphylococcus aureus* with OEO, which significantly reduced the number of bacterial cells, and the surface of bacterial cells collapsed and atrophied. Dutra et al. [53] treated *Alicyclobacillus* spp. with OEO and showed cell wall destruction and integrity deformation. This may be because OEO affected the membrane permeability and membrane integrity of *V. vulnificus* and caused the intracellular content to flow out, leading to the original morphological change of bacteria, and eventually causing irreversible damage to the bacteria.

*V. vulnificus* contamination in oysters is high concentration and high risk [54]. Thus, an assay in fresh oysters was taken, and results of our study showed that the number of bacteria decreased by 48.2% after 10 h of treatment with 0.09% OEO, which proved that OEO could effectively control *V. vulnificus* in oysters (Figure 10). Similarly, Mahmoud [55] showed that treatment with 500, 300, 150 mg/mL grape seed extract, citric acid or lactic acid solutions significantly reduced the initial inherent microbiota in fresh shucked oysters (*p* < 0.05) during 20 days of storage at 4 °C. Meanwhile, Hernandez et al. [56] discussed the microbial control ability of OEO in air-dried beef, and the results showed that OEO significantly reduced the number of *Salmonella enteritidis* and *E. coli* in air dried beef. The current study showed that the inhibitory effect of OEO against *V. vulnificus* was better in APW medium (Figure 2) than in the fresh oyster medium (Figure 10). This may be due to the fact that fresh oysters contain various amino acids, glycogens, active trace elements and small molecular compounds that could inhibit the antibacterial activity of OEO. Consequently, some scholars have proposed possible solutions to better employ the antibacterial properties of OEO in complex media. Both the OEO Pickering extrusion with good biocompatibility and chemical stability prepared by Zhou et al. [57] and poly (hydroxybutyrate-co-hydroxyvalerate)-based nanocomposites prepared by Costa et al. [58] for the packaging of antibacterial active food containing OEO can effectively inhibit the growth of tested Gram-positive and Gram-negative bacteria. In the future, the preparation of OEO-Pickering emulsion and the use of nano-embedding technology will be explored to further improve the antibacterial activity of OEO in food, and sensory changes will be comprehensively explored through new antimicrobial methods based on OEO.

**Figure 11:**
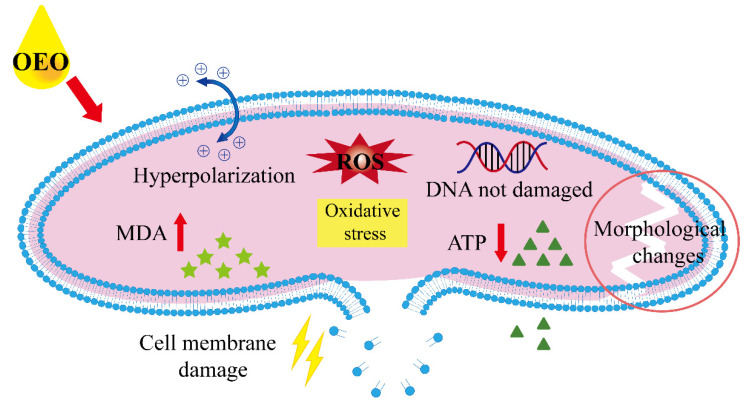
Bactericidal mechanism of OEO against *V. vulnificus*.

In conclusion, our study reported the good antibacterial activity and mode of action of OEO against *V. vulnificus*, as presented in Figure 11. OEO induces the rise of ROS levels to trigger the lipid peroxidation of cell membrane, so as to destroy the permeability and integrity of cell membrane and then change the bacterial morphology. Membrane hyperpolarization and intracellular ATP concentration decreased, MDA concentration increased, and CLSM and FESEM further confirmed membrane damage. Moreover, 0.06% and 0.09% OEO reduced the number of bacteria by 29.6% and 48.2%, respectively, showing good bactericidal ability against *V. vulnificus* in fresh oysters. Therefore, OEO has potential for use in oysters and other seafoods industry as a natural antibacterial agent to reduce the risk of *V. vulnificus* infection. Therefore, OEO has the potential to be used as a natural antibacterial agent in the oyster and other seafood industries to reduce the risk of *V. vulnificus* infection.

## Figures and Tables

**Table 1:** Minimum inhibitory concentration (MIC) and minimum bactericidal concentration (MBC) values of *Origanum vulgare* L. (OEO) against four strains of *Vibrio vulnificus.*

Strain	MIC (μL/mL)	MBC (μL/mL)
ATCC 27562	0.06	0.06
VV02	0.08	0.08
VV15	0.15	0.15
VV20	0.08	0.08

## Supplementary Materials

The following supporting information can be downloaded at: https://www.mdpi.com/article/10.3390/foods11030403/s1, Figure S1: The relationship between the concentrations of ATP and relative luminescence units.

## Abbreviation

OEO	Oregano essential oil
*V. vulnificus*	*Vibrio vulnificus*
MIC	Minimum inhibitory concentration
MBC	Minimum bactericidal concentration
ROS	Reactive oxygen species
MDA	Malondialdehyde
DMSO	Dimethyl sulfoxide
APW	Alkaline peptone water
TSA	Tryptone soya agar
PBS	Phosphate-buffered saline
PBS	Phosphate-buffered saline
PI	Propidium iodide
CLSM	Confocal laser scanning microscopy
FESEM	Field-emission scanning electron microscopy
OD	Optical density
SD	Standard deviation
