# Supplementary figures and images for: Antibacterial Effect of Oregano Essential Oil against Vibrio vulnificus and Its Mechanism
Source: Foods. 2022 Jan 30;11(3):403. doi: 10.3390/foods11030403 (PMC8834123; doi:10.3390/foods11030403)

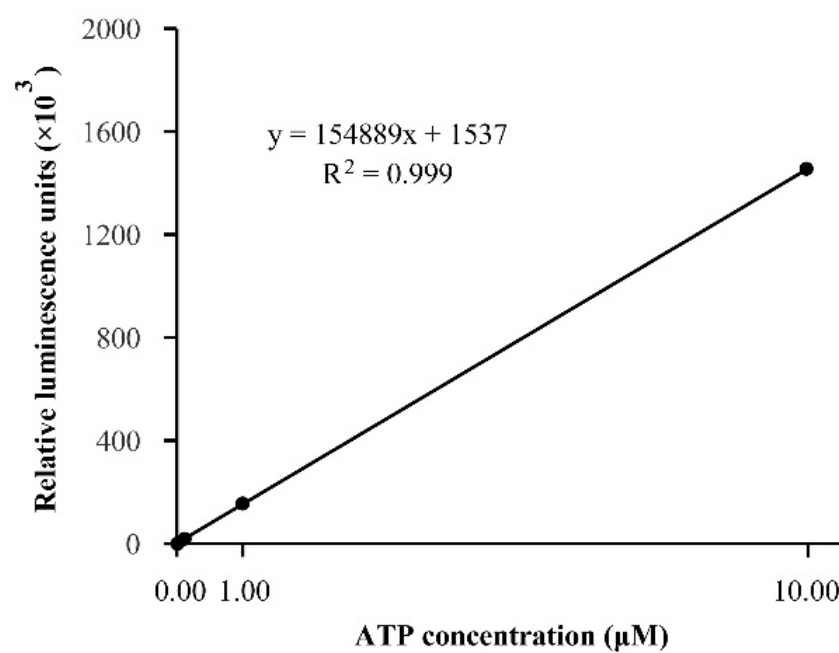

**Figure S1.** The relationship between the concentrations of ATP and relative luminescence units.

Supplement: Supplementary file 1 [file foods-11-00403-s001.zip › foods-1489395-supplementary.pdf]
